# Aging: Dial M for Mitochondria

**DOI:** 10.18632/aging.100118

**Published:** 2010-01-26

**Authors:** Jae H. Hur, Jaehyoung Cho, David W. Walker

**Affiliations:** ^1^ Department of Physiological Science, University of California, Los Angeles, Los Angeles, CA 90095, USA; ^2^ Molecular Biology Institute, University of California, Los Angeles, Los Angeles, CA 90095, USA

**Keywords:** Drosophila, longevity, C. elegans, respiratory chain

## Abstract

A major goal of aging research is to identify interventions that prolong lifespan in distantly related organisms.  In recent years, genetic studies in both nematodes and rodents have reported that moderate inactivation of genes important for mitochondrial electron transport chain (ETC) function can promote longevity.  We performed an RNAi screen to probe the role of ETC components in modulating lifespan in the fruit flyDrosophila melanogaster.  In this Research Perspective, we discuss our findings and how they may relate to similar studies in worms and mice.

Aging is characterized by a general decline in physiological function that leads to morbidity and eventually, mortality.   In recent years, significant progress has been made in our understanding of the nature of these changes at the molecular level. Due to the descriptive nature of these studies however,it has proven difficult to infer the relative importance of the many processes that contribute to aging.  Alterations in mitochondrial energy metabolism may be particularly important because it is central to metabolism a vital process that affects every aspect of cellular function.  Moreover, genetic studies in a number of model organisms have reported that inactivation of genes important for mitochondrial function can have profound effects on lifespan.

In this Perspective, we discuss our recent findings in the fruit fly *Drosophila* and how they relate to studies in other model organisms, including mammals.

## Age-related changes in mitochondrial function

Defects in mitochondrial electron transport chain (ETC function and their effects on aging and age-related disease have been widely reported [1,2].  Aged mammalian tissues show a decreased capacity to produce ATP and the impairment of mitochondrial function has been attributed to decreased rates of electron transfer by the selectively diminished activities of complexes I and IV [3].  In addition to decreased electron transfer and oxygen uptake, dysfunctional mitochondria in aged rodents are characterized by increased oxidation products of phospholipids, proteins andDNA, decreased membrane potential, and increased size and fragility [4].  These features of the aging process may be conserved across the animal kingdom.  For example, in *Drosophila* aging is also associated with changes in mitochondrial structure [5,6] and a decline in mitochondrial function [7].  As in mammals, complex IV activity appears to be particularly vulnerable to both aging [7] and oxidative stress [6] in flies. These observations have lead to the concept of the "vicious cycle" in which an initial ROS-induced impairment of mitochondria leads to increased oxidant production that, in turn, leads to further mitochondrial damage [8].

Another possible explanation behind the age-related decline in ETC function is the decline in expression of nuclear-encoded components of the ETC.  In one study, the authors compared microarray data between two organisms (*Caenorhabditis elegans* and *Drosophila*) as they aged in an effort to obtain a consensus aging transcriptsome. In both species, there was a significant decrease in a large set of genes involved in ATP synthesis and mitochondrial respiration [9].  Similar studies have identified age-related declines in the expression of genes involved in ETC function in both humans and mice [10,11].  At present, it is not known if these changes in gene expression are a cause, consequence or correlate of the aging process.

## Inactivation of genes important for ETC function

## Caenorhabditis elegans

The link between mitochondrial energy metabolism and longevity has been most extensively studied in the nematode *C. elegans*. Numerous studies have demonstrated that direct disruption of the mitochondrial electron transport chain (ETC) can produce large effects on lifespan.  Although short-lived mutants are often considered less informative than long-lived mutants as they may result from novel pathologies unrelated to normal aging, the *mev-1* mutant may be a notable exception.  This short-lived mutant carries a mutation in subunit C of mitochondrial complex II [12] and displays elevated ROS production [13] which has been proposed to be an important determinant of lifespan [8].

One of the first demonstrations that impaired ETC function could increase lifespan was the isolation and characterization of a long-lived mutant with a mutation in the iron sulfur protein (*isp-1*) of mitochondrial complex III [14].  The long-lived *clk-1* mutant lacks an enzyme required in the biosynthesis of ubiquinone, also known as coenzyme Q, an important electron acceptor for both complex I- and II-dependent respiration [15].  These results have been greatly expanded by a number of large-scale RNA-interference (RNAi) screens that have demonstrated that reducing the level of various components of respiratory chain complexes I, III, IV, or V results in longer-lived worms [16-20].  In addition, feeding worms a diet of respiratory deficient bacteria is also sufficient to extend lifespan [21,22].

When interpreting these findings, it may prove important to consider certain aspects of nematode ecology and physiology [23,24].  Worms make their living in the soil and, if given a choice, prefer hypoxic conditions [25], under which they can survive for remarkably long periods [26].  Physiologically, this may be enabled by an anaerobic energy-generating pathway, not found in mammals, involving reverse electron transfer via fumarate reductase and malate dismutation [27]. It may prove telling that the phenotypic consequences of elevated mitochondrial oxidative stress differ in worms and other animals.  Deletion of the mitochondrial superoxide dismutase 2 (*sod2*) results in very severe shortening of lifespan in both flies [28] and mice [29].  In contrast, *sod-2* deficiency in *C. elegans* has been reported to increase longevity in one study [30] and have negligible impact on longevity in another study [31].  Therefore, it is possible that the physiological and phenotypic consequences of ETC dysfunction may be different in worms compared to those in other animals. With this in mind, we thought it important to examine the impact of moderate inactivation of ETC genes in a different model organism.

## Drosophila melanogaster

The isolation and characterization of a *Drosophila *mutant with a defect in the iron-sulfur subunit (*sdhB*) of complex II was the first study to directly investigate the effects of decreased ETC function on fly longevity [32]. Interestingly, the biochemical and phenotypic consequences of complex II deficiency in flies are very similar to complex II (*mev-1*) deficiency in worms.  Specifically, our biochemical data indicate that SDHB is critical in preventing electron leakage from complex II, so that mutant animals suffer from increased oxidative stress and, as a result, are highly sensitive to oxygen and die rapidly [32].  The fact that inactivation of complex II had similar phenotypic effects in flies and worms led us to question the consequences of moderate inactivation of other ETC genes in the fly.

By happy coincidence the generation of a genome-wide library of *Drosophila* RNAi transgenes [33] was reported shortly before one of us (D.W.W) moved to UCLA to start his independent research group.  This technical advance allowed us to systematically inactivate a large fraction of nuclear encoded ETC genes in living flies and study their impact on longevity [34].  Not surprisingly, we observed that many of the RNAi lines produced larval lethality or shortened adult longevity.  However, just like in the worm, we observed that RNAi-inactivation of certain ETC genes resulted in enhanced longevity [34].   A major conclusion of our study is that despite more than 600 million years of separate evolution, partial inactivation of certain ETC genes can promote longevity in both fies and worms.  Furthermore, we observed that neuronal-specific RNAi mediated knock-down of certain ETC genes was sufficient to extend lifespan.  The effects on longevity of tissue-specific ETC gene inactivations have not yet been reported in *C. elegans*.

Shortly after publication of the results of the RNAi screen, we reported that genetic and pharmacological treatments that target complex V affect lifespan in a nutrient-dependent manner [35].  In an independent study, similar observations were reported for complexes I and IV [36].  Together these findings strongly suggest that altered ETC activity plays a critical role in dietary restriction-mediated longevity in the fly.

## Mammals

In humans, mitochondrial defects have been implicated in a wide range of life-shortening degenerative diseases [2,37].  However, despite considerable progress in elucidating the underlying genetic defects, the molecular pathogenesis events linking the mutated gene to the observed clinical phenotype are poorly understood.  Unfortunately, relatively few rodent models of ETC deficiency have been reported.  The generation of a mouse lacking subunit D of complex II (SDHD) was reported as the first rodent model lacking a protein of the ETC [38].  Animals without an intact copy of *sdhD* die at early embryonic stages, while heterozygotes display complex II deficiency without alterations in body weight or major physiological dysfunction.  More recently, it was reported that inactivation of the *Ndufs4* gene, which encodes an 18 kDa subunit of complex I, leads to an early-onset (5 weeks) encephalomyopathy [39].

There have also been a number of reports indicating that certain genetic manipulations that impair ETC function can protect against oxidative stress, neurodegeneration, obesity, and diabetes, and prolong longevity in mice.  For example, tissue-specific deletion of apoptosis inducing factor (AIF) leads to reduced ETC function and was shown to protect mice against both diabetes and obesity [40,41].  Reduced activity of MCLK1, a mitochondrial enzyme necessary for ubiquinone biosynthesis, leads to a severe reduction of ETC function and a substantial increase in lifespan with no trade-off in growth or fertility[42,43].  Finally, mice carrying a disruption in SURF1, a putative complex IV assembly factor, display a complex IV biochemical defect, markedly prolonged longevity and complete protection from kainic acid-induced neurotoxicity [44].  It should be noted that these studies do not directly manipulate ETC gene activity. However, they clearly demonstrate that not all perturbations of ETC function in mammals are deleterious.   A recent study that did directly manipulate an ETC gene supports the idea that under specific conditions, ETC inhibition can reduce oxidative stress and neurodegeneration in mammals. Targeted deletion of a complex IV subunit (COX10) in neurons was shown to decrease both oxidative stress and amyloid plaque formation in a mouse model of Alzheimer's disease [45].

## Electron transport chain activity and longevity: less is more?

Taken together, the work in flies, mice and the large number of long-lived ETC mutant worms clearly demonstrates that decreased expression of certain ETC genes is an evolutionarily conserved mode of lifespan extension.  However, the underlying mechanisms remain poorly understood.  Our own work indicates that ETC gene manipulations that prolong lifespan are not necessarily associated with obvious energetic or physiological trade-offs.  We identified five ETC gene hypomorphs associated with increased longevity.  However, only two of the ETC gene knock-downs conferred detectable decreases in the abundance of fully assembled respiratory complexes.  In addition, none of the five ETC gene perturbations resulted in a decrease in ATP levels.   An important next step will be a careful and detailed analysis of different aspects of respiratory chain function in long-lived flies with reduced expression of ETC genes.

## Summary/Conclusions

Increased lifespan conferred byRNAi of mitochondrial respiratory components is not limited to nematodes but is also true for flies [34].   In addition, there is a growing body of data in mice, linking moderate respiratory chain dysfunction with enhanced longevity.  Thus this mode of lifespan extension has the potential to be generally applicable to animals, as is the case for dietary restriction and altered insulin/IGF-1 signaling.  There is, therefore, an urgent need for a better understanding of this ‘Public' mechanism of aging.

A challenge that we find particularly pressing is to reconcile our results with the large number of studies that report an age-related decline in ETC gene expression and activity.  If ETC activity decreases as a function of age, then it appears counterintuitive that RNAi knock-down of ETC genes would promote longevity.  In contrast, we might speculate that strategies to increase respiratory chain function may prove effective in retarding the aging process. In yeast, manipulations that increase respiration are associated with increased longevity [46,47]. Critically, experimental manipulations that directly increase the activity of respiratory enzymes in metazoans have been lacking.   And so, the consequences of increased respiratory chain activity in animals remain largely unexplored.   It is an exciting time to be studying the role of ETC activity in modulating animal aging.
